# Nanoaggregate-Based Innovative Electrochemiluminescence Sensors for Foodborne Contaminant Analysis

**DOI:** 10.3390/bios16010006

**Published:** 2025-12-22

**Authors:** Tingting Han, Jinyang Zhuang, Yueling Lu, Jianhong Xu, Jun-Jie Zhu

**Affiliations:** 1 Jiangsu Key Laboratory of Food Quality and Safety, Collaborative Innovation Center for Modern Grain Circulation and Safety, Institute of Food Safety and Nutrition, Jiangsu Academy of Agricultural Sciences, Nanjing 210014, China; 20230027@jaas.ac.cn (T.H.); 2023210107@jou.edu.cn (Y.L.); 2 Key Laboratory of Quality and Safety of Cereals and Their Products, State Administration for Market Regulation, Quality and Safety Institute of Agricultural Products, Heilongjiang Academy of Agricultural Sciences, Harbin 150086, China; 3 State Key Laboratory of Analytical Chemistry for Life Science, School of Chemistry and Chemical Engineering, Nanjing University, Nanjing 210023, China; jinyangzhuang@smail.nju.edu.cn

**Keywords:** electrochemiluminescence, nanoaggregates, foodborne contaminants, sensors, food safety

## Abstract

The pervasive presence of foodborne contaminants in foods poses a significant global threat, contributing to various foodborne diseases and food safety issues. Therefore, developing rapid, sensitive, and universal detection methods for them is essential to ensure public health and food safety. Electrochemiluminescence (ECL) sensors, particularly those incorporating innovative nanoaggregates, have been widely used to detect related contaminant residues in foodstuffs owing to their superior sensitivity and low background signals. This review summarizes recent advances in nanoaggregate-based novel ECL sensors for detecting a wide range of contaminants, with emphasis on their fundamentals and representative applications. This area has not yet been comprehensively covered in the existing literature. The current challenges and emerging trends for next-generation ECL sensors based on nanoaggregates in food safety monitoring are also discussed.

## 1. Introduction

Foodborne diseases have garnered increasing global attention in recent years, because they can induce a series of food safety issues that increase social and economic burdens [1]. Foodborne diseases are mainly caused by different food contaminants like pathogens, mycotoxins, pesticide residues, antibiotics, heavy metal ions, and illegal additives [2]. To effectively prevent the occurrence of foodborne diseases and ensure food safety, it is indispensable to analyze these possible contaminants in food and related products. So far, a vast number of analytical technologies have been developed for the detection of these foodborne contaminants such as mass spectrometry [3], high-performance liquid chromatography [4], fluorescence (FL) [5], electrochemistry [6], surface-enhanced Raman spectroscopy [7], and photoelectrochemistry (PEC) [8]. However, there are still some problems with these technologies as result of the complexity of the food matrix and the low abundance of these contaminants in foods, involving cumbersome sample preparation, pronounced background interference, and limited sensitivity and accuracy. Consequently, the development of rapid, sensitive, and reliable analytical methods to analyze foodborne contaminants in foods and related products is urgently required.

Electrochemiluminescence (ECL) is essentially a type of chemiluminescence triggered by electrochemical reactions [9,10]. Due to its high sensitivity, minimal background signal, wide dynamic range, and operational simplicity, ECL has been widely regarded as a promising analytical tool in foodborne contaminant detection [11]. To effectively boost its analytical capabilities for low-abundance foodborne contaminants within complex food matrices, all kinds of nanoaggregates, including metal nanoparticles, quantum dots (QDs), polymer dots (Pdots), metal nanoclusters (NCs), aggregation-induced emission (AIE)-based aggregates, and hydrogen-bonded organic frameworks (HOFs), have emerged and been widely incorporated into ECL biosensors [12]. These nanoaggregates are provided with different functions and roles in ECL applications, which not only work as new-type ECL nano-emitters for target signal generation, but also function as nanocarriers for loading luminophores and catalysts for facilitating the decomposition of co-reactants, greatly improving signal responses and detection sensitivity [13]. Leveraging these functional nanoaggregates, the developed ECL sensors have achieved significant achievements in both sensitivity and selectivity in detecting foodborne contaminants in recent years [14]. Advancing this connection, a comprehensive summary of ECL sensors based on nanoaggregates will guide the evolution of next-generation monitoring solutions from regulator standards to commercial detectors and warning systems to better control foodborne contaminant residues in foodstuffs and ultimately mitigate risks to human health and food security.

**Scheme 1:**
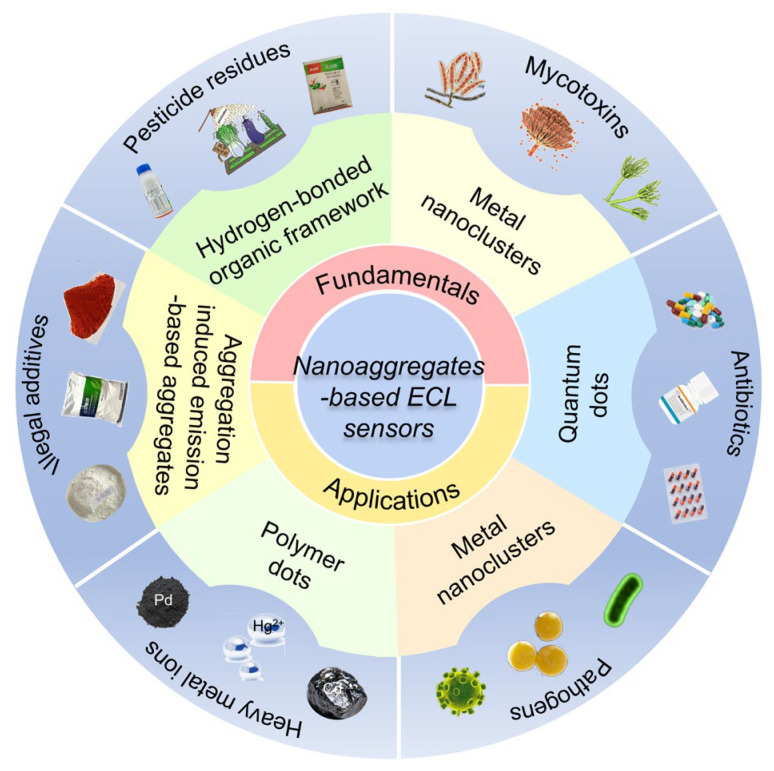
Schematic illustration of nanoaggregate-based ECL sensors for determining foodborne contaminants.

Although the past few decades have witnessed a surge in research and reviews focusing on nanoaggregate-based ECL sensors for foodborne contaminant analysis [15,16], to the best of our knowledge, no comprehensive review has been dedicated to ECL biosensors based on nanoaggregates in foodborne contaminant determination. Therefore, a timely and comprehensive review of nanoaggregate-based ECL sensing strategies for profiling foodborne contaminants is of great significance for inspiring relevant researchers to explore innovative ECL sensors through continued refinement and development. In this review (Scheme 1), we review the recent progress in nanoaggregate-based ECL sensors for detecting foodborne contaminants, outlining their development trajectory from the fundamentals to representative applications. Lastly, present challenges and future trends in the development and application of advanced nanoaggregate-based ECL sensors for foodborne contaminants are also discussed. This review aims to provide guidance and inspiration for future studies focused on ECL biosensors and their supervisory systems related to foodborne contaminants, paving the way for advancements in food safety and public health protection.

## 2. Fundamentals of Nanoaggregate-Based ECL Sensors

### 2.1. Working Principle

The operational principle of nanoaggregate-based ECL sensors relies on an integrated system that comprises a recognition element and a transducer for identifying food contaminants in foodstuffs. The recognition element, like an antibody, aptamer, or molecularly imprinted polymer (MIP), is primarily used to determine the presence and content of targets within a sample. The transducer transforms the binding event identified by the recognition element into a measurable output signal (e.g., current or ECL intensity), specifically by determining the ECL signal produced from redox reactions at the electrode that correlates with the target analyte’s presence and concentration. The recent developments in various ECL sensors have spurred considerable interest in their applications for quantitative and semi-quantitative analysis, driving the establishment of numerous ECL sensing approaches for a wide range of analytes [17,18]. Nanoaggregate-based ECL sensing technologies operate on the principle of utilizing nanomaterial assemblies with different functionalities that participate directly or indirectly in the ECL process via chemical modification and ECL intensity variation, enabling the detection of foodborne contaminants. These reagents, like active or/and recognition elements, are integral to the ECL sensing process used in foodborne contaminant detection. Among them, nanoaggregates as active elements have enabled significant enhancements in sensitivity and signal response through their unique functionalities, thus playing a key role in sensor performance.

### 2.2. Nanoaggregates as the Active Elements of ECL Sensors

Nanoaggregates are described as stable assemblies with dimensions in the nanoscale range, which are formed by the spontaneous or induced aggregation of multiple nanoscale basic units (e.g., nanoparticles, small molecules, polymer chains, etc.) via non-covalent interactions like van der Waals forces, hydrogen bonding, π-π stacking, and so on [19]. With the benefits of unique physicochemical properties like surface effect, AIE effect, and quantum size effect, nanoaggregates exhibit exceptional electrochemical and optical characteristics, such as a high surface-to-volume ratio, superior electron transport capability, and good biocompatibility [20]. These intrinsic properties make them ideal active components in ECL sensors, showing wide applications in catalysis, biomedical, and sensing fields [21,22]. As such, nanoaggregates can be engineered to play diverse roles within ECL sensors: (1) they can serve as nanocarriers for luminescent reactants and recognition elements (e.g., antibodies, aptamers) to contribute to enhanced ECL signals and improved specificity and detection sensitivity; (2) they can facilitate reaction kinetics as nano-catalysts to obtain higher ECL outputs; (3) they can act as ECL quenchers to enable precise “control” of ECL signals, greatly improving the sensors’ sensitivity and selectiveness; (4) they also function as ECL nano-emitters or co-reactants in ECL sensors, participating in reactions with other components to generate observable output signals. In short, nanoaggregates as multifunctional active ingredients provide powerful guarantees for optimizing ECL sensors’ sensibility, specificity, and speed of response, which has rendered the use of nanoaggregate-based ECL sensors a prominent strategy for highly sensitive analyses of trace analytes. As a consequence, we outline several popular nanoaggregates, including metal nanoparticles, QDs, Pdots, metal nanoclusters, AIE-based aggregates, and HOFs, to highlight their distinct functional roles in practical ECL sensing applications.

Metal nanoparticles (such as AuNPs, AgNPs) exhibit outstanding catalytic activity in ECL reaction processes because of their large surface area, elevated surface energy, distinctive electronic structures, and the reactivity of their surface atoms [23]. Furthermore, they also have localized surface plasmon resonance (LSPR), which remarkably improves ECL efficiency through strong coupling interactions between free electrons in specified metals and luminophores [24]. For instance, Sun’s group [25] reported an ultrasensitive ECL aptasensor with AgNPs as an effective catalyzator for probing kanamycin (KAN) in milk in view of the high specificity of the KAN–aptamer interaction. Mei et al. [26] constructed a paper-based ECL platform based on self-enhanced Zn-MOF and the LSPR effect of AuNPs for deoxynivalenol (DON) analysis through specific binding to its matched aptamer.

QDs are defined as semiconductor nanocrystals (2–20 nm) typically composed of II-VI or III-V group elements, which are highly attractive for development in ECL analysis due to their size-tunable luminescence, high quantum efficiency, and excellent resistance to photobleaching [27]. In these applications, they function not only as emitters and co-reactants to generate signals, but also as nano-catalysts to enhance the ECL reaction efficiency for the intensified outputs. Therefore, Jiang and his colleagues [28] constructed a novel solid-state ECL sensing platform based on 0D g-C_3_N_4_ QDs@3D graphene hydrogel (CNGH) nanocomposites as the ECL nanoemitters for ultrasensitive KAN detection, resulting from the highly specific target–aptamer interaction. Kamyabi et al. [29] reported a robust nonenzymatic ECL sensor using ruthenium nanoparticles (Ru NPs), being a popular ECL probe, and boron nitride QDs (BNQDs) as an efficient co-reactant for supersensitive detection of trace diazinon in real samples. In addition, Wang et al. [30] developed a self-enhanced MIP-ECL sensor based on M-Ag@MoS_2_ QDs as both ECL emitters and coreactants for thiabendazole (TBZ) measurement in oranges, potatoes, and grapes due to the specific recognition between imprinted cavities in MIPs and the target.

Different from traditional QDs, Pdots are a category of nanoscale luminescent materials formed by the self-assembly/precipitation of conjugated or functional polymers, which have been widely developed as effective ECL signal probes in clinical diagnosis and food safety analysis because of their ultra-high brightness, excellent photostability, outstanding biocompatibility, and ease of function [31]. Chen et al. [32] established an ultrasensitive ECL resonance energy transfer (ECL-RET) sensor using TPE-based AIE-active Pdots and black hole quencher (BHQ) as the donor–acceptor pair for accurate and quantitative detection of arsenite (As(III)) in rice by specifically binding to its corresponding aptamer. He et al. [33] designed a novel ratiometric ECL sensing platform coupled with potential-tunable poly [9,9-bis(3′-(N,N-dimethylamino)propyl)-2,7-fluorene]-alt-2,7-(9,9-dioctylfluorene)] nanoparticles (PFN NPs) with satisfactory anodic dual-ECL signals for detecting organophosphorus pesticides (OPs) in vegetables, apples, and water as a result of the OP-induced suppression of hydrogen peroxide (H_2_O_2_) generation via AChE inhibition.

Unlike conventional nanoaggregates, metal nanoclusters (e.g., AuNCs, CuNCs, AgNCs, etc) feature an atomically precise structure composed of a defined number of metal atoms and a single layer of organic ligands, which have become widely studied for new ECL mechanism exploration and biosensing due to their atomically precise size, compositional diversity, and stable optoelectronic properties [34]. They not only work as ECL nanoprobes for obtaining satisfactory ECL signals, but also serve as fresh co-reactants for amplifying luminophores’ ECL intensities, contributing to improvements in the sensitivity of ECL assays [35]. Han’s group manufactured [36] an ultrasensitive label-free ECL immunosensor armed with AuNCs in chitosan (AuNCs@CS) nanogels as an efficient ECL nanoprobe for selective aflatoxin B1 (AFB1) detection in Chinese herbal medicine owing to their high specificity in antigen–antibody recognition. Peng et al. [37] constructed a new self-calibrating ECL immunosensing platform using RuSiNPs as a dual-signal probe and CuNCs as the coreactant for sensitive okadaic acid (OA) detection in oysters, as a consequence of their highly precise matching between antigen and antibody.

In addition, AIE-based aggregates are a type of nanoaggregates formed by the self-assembly of AIE-active small molecules (such as tetraphenylethylene (TPE) and its derivatives) via weak interactions, which propels rapid development in biomedical and biosensing fields on account of their high AIE efficiency, excellent stability, and good environmental tolerance [38]. AIE-based aggregates are commonly used as enhanced ECL luminophores to ensure the efficiency of ECL signals and the accuracy of detection results. In this regard, Lv and her collaborators [39] reported a label-free ECL immunosensor with 9,10-Diphenylanthracene cubic nanoparticles (DPA CNPs) as a stable and intense aggregation-induced electrochemiluminescence (AIECL) emitter for the ultrasensitive detection of AFB1 in walnut. Afterward, Wang’s group [40] fabricated a highly sensitive ECL aptasensor combining TPE NAs as the AIECL nanoprobe with the catalyzed hairpin assembly (CHA) amplification strategy for zearalenone (ZEN) assays in corn and wheat flour.

HOFs are a class of porous crystalline materials self-assembled from organic molecular building blocks through hydrogen bonding interactions, which play a pivotal role in many cutting-edge research directions, from gas storage and separation to catalysis and biomedicine, due to their highly adjustable pore structures, abundant functional sites, and distinctive network-like structures [41]. In ECL research, these HOFs are mainly used as emerging ECL nano-emitters for generating intensified ECL emissions. Herein, Li et al. [42] designed a novel ECL and PEC dual-mode sensor by using HOF-101 as an excellent dual-signal probe and polydopamine nanoparticles (PDAs) as the quenchers for the sensitive detection and visual analysis of oxytetracycline (OTC) via its specific binding to complementary aptamers to form dual-aptamer sandwich structures, which achieved limits of detection (LOD) as low as 0.04 pM and 0.3 pM.

### 2.3. Sensing Patterns

Leveraging the combined advantages of nanoaggregates and ECL technologies, nanoaggregate-based ECL sensors have seen extensive development. Among them, these sensors employ various sensing patterns for foodborne contaminant analysis, which were categorized as follows: “signal on”, “signal off”, ratiometric, and multimodal sensing modes.

#### 2.3.1. “Signal On” Sensing Mode

**Figure 1:**
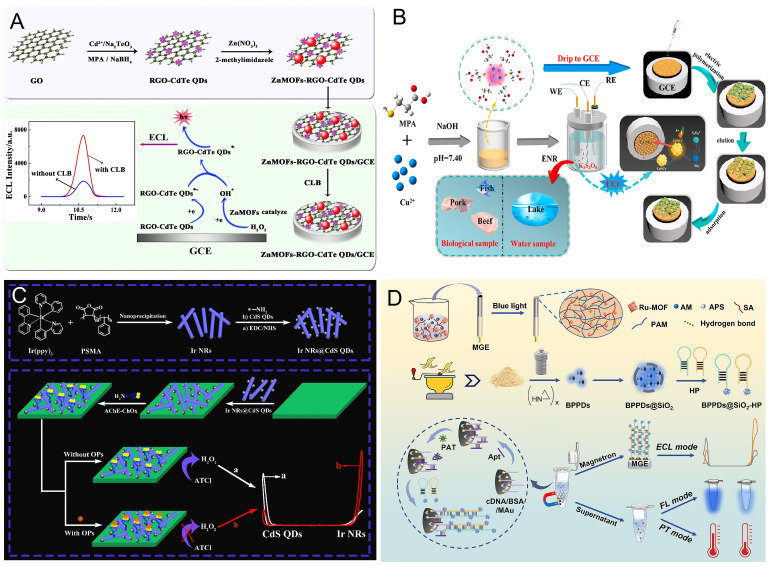
(**A**) Schematic illustration of a “signal on” ECL biosensor with ZnMOFs-RGO-CdTe QD composites for CLB detection. Reprinted with permission from Ref. [43]. Copyright 2018, Springer. (**B**) Diagram of a highly sensitive “signal off” CuNCs-based MIP-ECL biosensor for ENR assays. Reprinted with permission from Ref. [45]. Copyright 2021, Elsevier. (**C**) Illustration of a potential-resolved ratiometric ECL sensor for OPs analysis. The red and white curves represent the ECL responses in (a) presence or (b) absence of OPs, respectively. Reprinted with permission from [48]. Copyright 2021, Elsevier. (**D**) Schematic diagram of a highly sensitive ECL/FL/PT multimodal aptasensor coupled with RuMOF@hydrogel and BPPDs for PAT detection. Reprinted with permission from Ref. [50]. Copyright 2024, Elsevier.

The ECL intensity is directly proportional to the analyte’s concentration, which is defined as a “signal-on” sensing mode. For example, Hu et al. [43] established a sensitive “signal on” ECL sensor with ZnMOFs-RGO-CdTe QDs hybrids for quantitative analysis of clenbuterol (CLB) in pork. In this hybrid, the RGO-supported CdTe QDs demonstrated strong ECL emission, which was further intensified by ZnMOFs that catalyzed the production of OH• radicals from the co-reactant H_2_O_2_. Upon theaddition of CLB, a concentration-dependent increase in ECL intensity was observed with increasing CLB concentration, because the target CLB was electro-oxidized into CLB• radicals that subsequently reacted with H_2_O_2_ to produce OH• species responsible for ECL signal amplification, consequently achieving good analytical performance in quantifying CLB along with a lower LOD of 0.1 pM (Figure 1A). Wei and her colleagues [44] reported a split “signal on” ECL aptasensor for the selective monitoring of trace KAN in milk and honey using cucurbit[7]uril@Try-MPA-AuNCs as an ECL probe and Zn-SnO_2_ NFs as an electrode substrate by means of highly specific response of KAN and its aptamer. The Zn-SnO_2_ NFs substrate could adsorb more Apt1 without ECL signal generation in the absence of KAN. After introducing KAN and Apt2-tagged cucurbit[7]uril@Try-MPA-AuNCs into the system, the sandwich structure was formed to obtain an intense ECL signal. This designed split aptasensor had highly sensitive measurement of KAN with an ultralow LOD of 32.90 fg/mL.

#### 2.3.2. “Signal Off” Sensing Mode

Conversely, the “signal-off” sensing mode is characterized by a decline in ECL response as the analyte concentration rises. Wang et al. [45] built a “signal off” MIP-ECL sensor with MPA-CuNCs as the AIECL nano-emitter for selectively detecting enrofloxacin (ENR) in biological and environmental analysis. The MPA-CuNCs with high ECL emission were immobilized on the electrode, followed by the electro-polymerization reaction of ENR as the template molecule and o-phenylenediamine (o-PD) as the functional monomer to fabricate MIPs with specific cavities. In the presence of ENR, the ECL intensity exhibited an inverse correlation with ENR concentrations, possibly due to the reaction between ENR and the S_2_O_8_^2−^ intermediates, which demonstrated good sensitivity in detecting ENR with a lower LOD of 27 pM (Figure 1B). Wu et al. [46] developed a new-type ECL sensor, employing the synergistic effects of Fe_3_CuO_4_ and CdS@ZnS QDs for ultrasensitive permethrin determination. In this system, Fe_3_CuO_4_ acts as both a nanocarrier and a co-reaction promoter, which allows for extensive Ru(bpy)_3_^2+^ adsorption and a consequent boost in ECL intensity. CdS@ZnS QDs were introduced as the co-reaction to further enhance their ECL signal. The ECL signal decreased as the concentration of permethrin increased, presenting excellent sensitivity in permethrin detection with a LOD as low as 3.3 pM.

#### 2.3.3. Ratiometric Sensing Mode

The ratiometric sensing mode, operating on dual-signal outputs (either “signal-on” or “signal-off”), has significantly improved the self-correction and anti-interference capacities of nanoaggregate-based ECL sensors, which has substantially elevated the detection accuracy and sensitivity of foodborne contaminant analysis in recent years. Wang’s group [47] reported a supersensitive potential-resolved ratiometric ECL sensor integrated with a Ru(bpy)_3_^2+^-doped trimetallic nanocube (Ru@Tri) as a dual-ECL signal probe for trace-level monitoring of patulin (PAT) in fruit products via a high specific interaction with its aptamer. In this study, the Ru@Tri composites served as both an ECL probe and a co-reaction promoter to yield an intense cathodic ECL signal with K_2_S_2_O_8_ as a result of Tri’s synergistic catalysis. In parallel, the anth-CQDs@SiO_2_ as the andic co-reactant would strengthen the andic ECL signal of Ru@Tri. Based on this phenomenon, the ratio of anodic to cathodic ECL signals showed a significant concentration-dependent increase with rising PAT levels. By leveraging the self-calibrating capability of this dual-signal output, the sensing platform can be effectively adapted for highly precise and quantitative analysis of trace PAT within the detection range of 0.0001–10 ng/mL, with its LOD calculated to be 0.05 pg/mL. Yang et al. [48] constructed an ultrasensitive ratiometric ECL sensor utilizing a combination of Ir nanorods and CdS QDs (Ir NRs@CdS QDs) as dual-polarity ECL nano-emitters for OP assays. Ir NRs@CdS QDs dropped on the electrode could immobilize acetylcholinesterase (AChE) and choline oxidase (ChOx) for H_2_O_2_ in situ production, which resulted in augmented cathodic ECL intensity and a diminished anodic ECL intensity of the Ir NRs@CdS QDs/TPA system without OPs. When OPs were introduced, ECL signal enhancement at the anode and ECL signal quenching at the cathode were observed, because OP-mediated suppression of AChE prevented H_2_O_2_ generation. On this basis, the constructed ratio ECL sensor for OP detection was developed, with a LOD of 1.67 pM (Figure 1C).

#### 2.3.4. Multimodal Sensing Mode

In addition to the above-mentioned sensing modes, multimodal sensing modes that integrate “signal-on” or “signal-off” ECL signals with auxiliary signal types (e.g., FL, EC, etc.) can endow ECL assays with superior selectivity, enhanced signal-to-noise ratio, and improved accuracy, thus showing considerable application prospects in sophisticated on-demand food analysis. Li et al. [49] designed a novel EC and ECL dual-mode aptasensor for sensitive AFB1 detection in peanuts based on the interactions between ferrocene (Fc) and nitrogen-doped graphene quantum dots (NGQDs)-Ru(bpy)_3_^2+^-doped silica (SiO_2_) nanoparticles (SiO_2_@Ru-NGQDs). In this work, AuNPs were assembled on a SiO_2_@Ru-NGQD-modified electrode and served as an immobilization matrix for complementary DNA (cDNA) via Au-S bonds. A Fc-marked aptamer (Fc-Apt) specific to AFB1 was then assembled, thereby enabling specific recognition and obtaining both an EC signal as well as an amplified ECL signal induced by Fc-distance dependent ECL response. After binding with Fc-Apt, the target AFB1 induced its dissociation from the electrode to significantly provoke the dual-quenching of the EC and ECL signals, ultimately realizing dual-mode sensing of AFB1 with high sensitivity. Thereafter, Wang’s group [50] proposed an innovative multi-mode aptasensing platform coupled with a Ru(bpy)_3_^2+^-based metal–organic framework-composited hydrogel (RuMOF@hydrogel) and SiO_2_-doped banana peel-derived carbonized polymer dots (BPPDs@SiO_2_) for detecting PAT in fresh fruits, fruit products, and infant foods on account of the aptamer’s ability to bind to PAT with high specificity. As exhibited in Figure 1D, the RuMOF@hydrogel demonstrated exceptional anodic and cathodic ECL emissions. The BPPDs@SiO_2_ as an anodic co-reactant served to amplify the ECL signal while boasting remarkable FL and photothermal (PT) characteristics, which labeled hairpin DNA (BPPDs@SiO_2_-HP) to serve as two signal probes. The addition of PTA initiated the HCR reaction to attach a massive BPPDs@SiO_2_-HP complex onto the MAu surface, leading to anodic ECL enhancement and cathodic ECL quenching to quantitatively detect PAT after magnetic separation. At the same time, the FL and PT responses of unbound BPPDs@SiO_2_-HP in the supernatant were negatively correlated with the PAT concentration. The designed ECL/FL/PT multimodal aptasensor for PAT assays showed satisfactory analytical performances, whose LODs were calculated as 2.5 fg/mL, 34 fg/mL, and 0.2 pg/mL, respectively.

## 3. Applications in Foodborne Contaminant Detection

The ongoing development of the food industry and the increasing diversification of food products have heightened the risk of contamination by microorganisms, toxins, pharmaceuticals, and additives during processing and production. Of grave concern is that these foodborne contaminants can cause multiple forms of neurological damage involving oxidative stress, impaired neuronal development, and altered neurotransmitter levels, posing a substantial threat to human and animal health [51,52]. As a result, food safety incidents resulting from such contaminants are increasingly a focus for experts in global health security and have become an urgent issue requiring resolution. ECL sensors are highly suitable for rapid on-site detection as a result of their straightforward results, ease of observation, and strong interference resistance [53,54]. Such capabilities, combined with the sensing technology’s high sensitivity, exceptional specificity, and ease of miniaturization, give it broad application potential in foodborne contaminant detection [55,56]. Until now, many nanoaggregate-based ECL sensors have been employed in various forms of foodborne contaminant detection, including pesticide residues, mycotoxins, antibiotics, pathogens, heavy metal ions, and illegal additives. In light of the foregoing, we will comprehensively review the applications of ECL sensors based on different nanoaggregates in detecting typical foodborne contaminants (Table 1).

### 3.1. Detection of Pesticide Residues

Pesticides are essential for controlling pests, diseases, and weeds, ensuring crop growth and improving agricultural productivity and quality [57,58]. With the growing types and usage of pesticides, non-standard or overuse of pesticides can lead to their residues persisting in agricultural products. These pesticide residues subsequently enter the food chain, potentially harming consumers’ health and polluting the ecological environment [59,60]. In response, the utilization of ECL sensors based on nanoaggregates as sensing platforms for monitoring a wide variety of pesticide residues has broad development prospects and has made major strides in this field.

**Figure 2:**
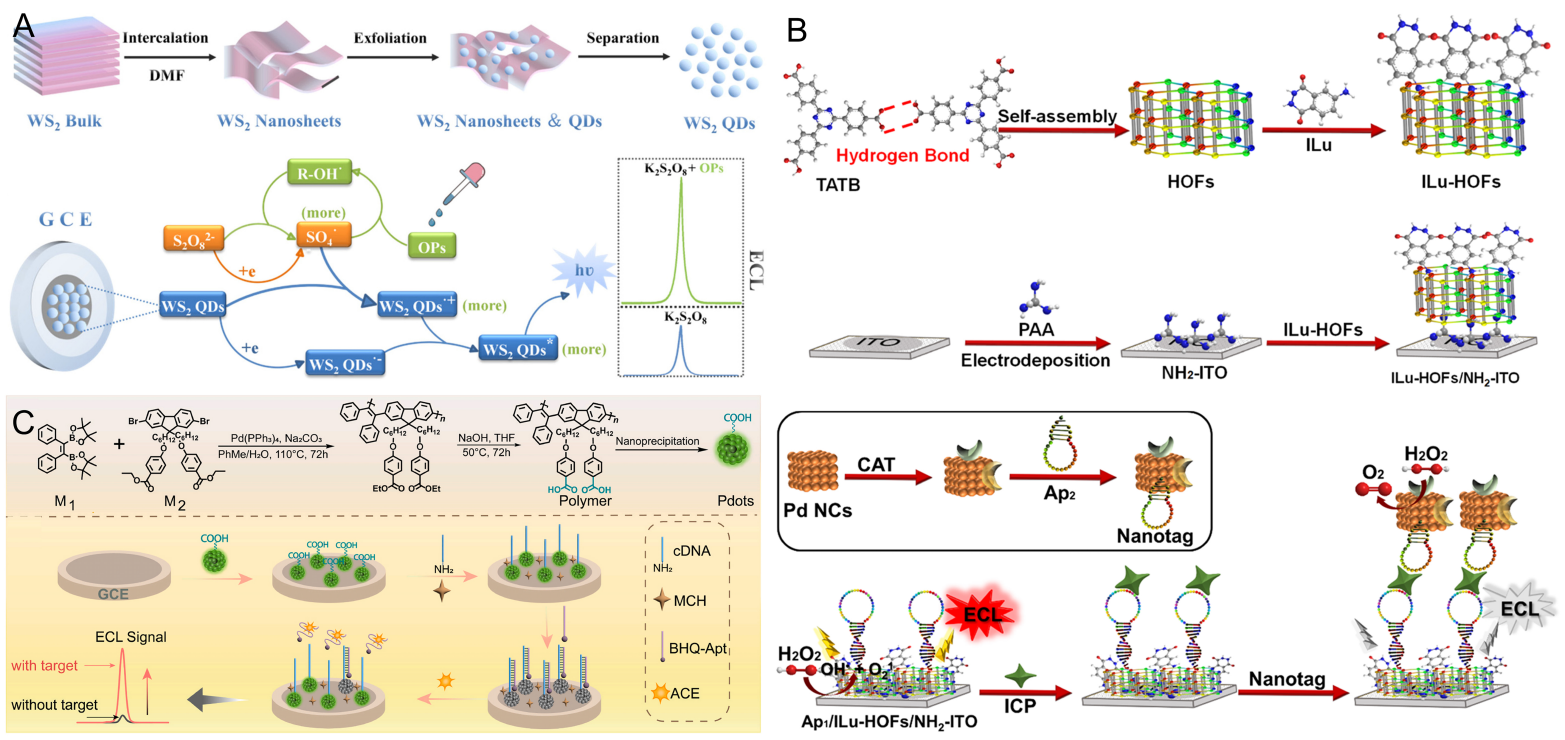
(**A**) Schematic diagram of a novel ECL sensor based on WS_2_ QDs for OPs detection. The light green and blue curves represent the ECL intensities with and without OPs, respectively. Reprinted with permission from Ref. [63]. Copyright 2024, Royal Society of Chemistry. (**B**) Diagram of a highly sensitive ECL biosensor with ILu-HOFs as the radical reactor for ICP analysis. Reprinted with permission from Ref. [65]. Copyright 2023, American Chemical Society. (**C**) Diagram of an ultrasensitive AIECL-RET aptasensor coupled with Pdots for determining ACE. The red and black curves represent the ECL intensities with and without ACE, respectively. The arrow represents the ECL intensities varied with ACE concentration. Reprinted with permission from Ref. [66]. Copyright 2025, Elsevier.

OPs mainly include malathion (MAT), isocarbophos (ICP), etc., which were once one of the most extensively used pesticide categories in global agricultural production due to their high toxicity and broad-spectrum insecticidal activity [61,62]. Nevertheless, their improper use has caused severe soil and water pollution. These pesticide residues persist in the environment and undergo bioaccumulation through the food chain, seriously threatening public health and safety. For instance, Sun et al. [63] constructed a sustainable enzyme-free tungsten disulfide QD (WS_2_ QDs)-based ECL biosensor to directly detect OPs in water and spinach. WS_2_ QDs with high ECL efficiency were prepared by ultrasonic hydrothermal-assisted liquid exfoliation of WS_2_ bulk and then modified on a GCE surface. Following the administration of OPs, their phosphate esters efficiently accelerated more free radical generation from the coreactant K_2_S_2_O_8_ to strengthen the ECL signal of the WS_2_ QDs under electrochemical stimulation, thus achieving direct ECL detection of OPs (Figure 2A). Tian’s group [64] proposed an ultrasensitive solid-state ECL sensing system based on Ru(bpy)_3_^2+^@AgNPs and TiO_2_@CdSe as the ECL tags for trace-level MAT monitoring in cucumber, cabbage, and spinach. In this system, TiO_2_@CdSe and Ru(bpy)_3_^2+^@AgNPs were co-modified onto the electrode for ECL signal maximization thanks to their cooperative effect with each other. The ECL signal dropped sharply upon the addition of MAT, which was explained by the fact that the adsorption of MAT onto the electrode interface caused an apparent drop in ECL intensity by blocking the active sites available for ECL reaction. Guided by the direct quenching mechanism, this proposed ECL sensor for detecting trace MAT demonstrated high selectivity and sensitivity with a LOD as low as 13 fM. In addition, Shen et al. [65] developed an aptamer “sandwich” ECL sensor based on isoluminol (ILu), HOFs, and catalase-linked palladium nanocubes (CAT-Pd NCs) for ultrasensitive ICP detection in lake water and cucumber juice samples owing to the high binding affinity between ICP and its aptamer. In this study, the Ilu-HOFs were prepared by linking ILu with HOFs that were self-assembled by 2,4,6-tris(4-carboxyphenyl)-1,3,5-triazine (TATB) and covalently assembled on an NH_2_-ITO electrode, which obtained high ECL efficiency in the presence of H_2_O_2_. This “sandwich-type” ECL sensing platform that was sequentially assembled from Ap1, ICP, and Ap2/CAT-PdNCs achieved a weakened ECL signal due to the in situ elimination of H_2_O_2_ by CAT-PdNCs, which sensitively detected ICP with a LOD of 0.4 pM (Figure 2B).

Benefiting from their high efficiency, low toxicity, and good systemic absorption abilities, which can be absorbed and transmitted by plants to effectively combat their hidden pests and pathogens, neonicotinoid pesticides like acetamiprid (ACE) have surpassed highly toxic OPs as the most prevalent new-type insecticides [67]. However, ACE, as a representative chloronicotinoid insecticide, is associated with potential hazards like carcinogenesis, sterility, endocrine dyscrasia, and fetal injury [68]. Founded on the above, Zhi et al. [69] built a sensitive ECL-RET aptasensor to accurately measure ACE residues in water based on Ag^+^@Eu-MOF/HOF and CdS@Au-cDNA as the donor–acceptor pairs. The prepared Ag^+^@Eu-MOF/HOF nanocomposites were fixed on the GCE surface, followed by a stepwise linkage of the ACE aptamer and CdS@Au-cDNA, which gave rise to a pronounced decline in the ECL signal as a result of the occurrence of the RET process. After the addition of ACE, the high-specificity binding between ACE and its aptamer triggered the release of CdS@Au-cDNA, consequently resulting in ECL signal restoration. The established sensor for detecting ACE displayed excellent sensitivity in the wide detection range of 0.1 nM-1.0 fM, accompanied by a lower LOD of 0.398 fM. Afterwards, Gao and his coworkers [66] engineered an ultrasensitive AIE-active Pdot-based ECL-RET aptasensing platform for ACE determination through highly specific binding to its corresponding aptamer. As shown in Figure 2C, the as-prepared Pdots with satisfactory AIE-ECL performance were linked with cDNA on GCE as the signal probe, which specifically hybridized with BHQ-Apt to quench ECL emission. The introduction of ACE recovered the ECL signal by dissociating the BHQ-Apt from the probe surface. The restoration of the ECL signal was proportional to the ACE concentration, enabling highly sensitive detection with a calculated LOD of 9.1 aM.

### 3.2. Detection of Mycotoxins

Mycotoxins are defined as secondary fungal metabolites generated by species like Aspergillus, Penicillium, and Fusarium during their growth, which can potentially induce serious health implications like teratogenicity, mutagenicity, carcinogenicity, and immunosuppression, greatly jeopardizing both human and animal well-being as well as causing huge economic losses [70,71]. In this case, a growing number of studies have been dedicated to designing and developing nanoaggregate-based ECL sensors for mycotoxin detection to provide a means for mitigating or circumventing the food safety risks posed by these contaminants [72].

**Figure 3:**
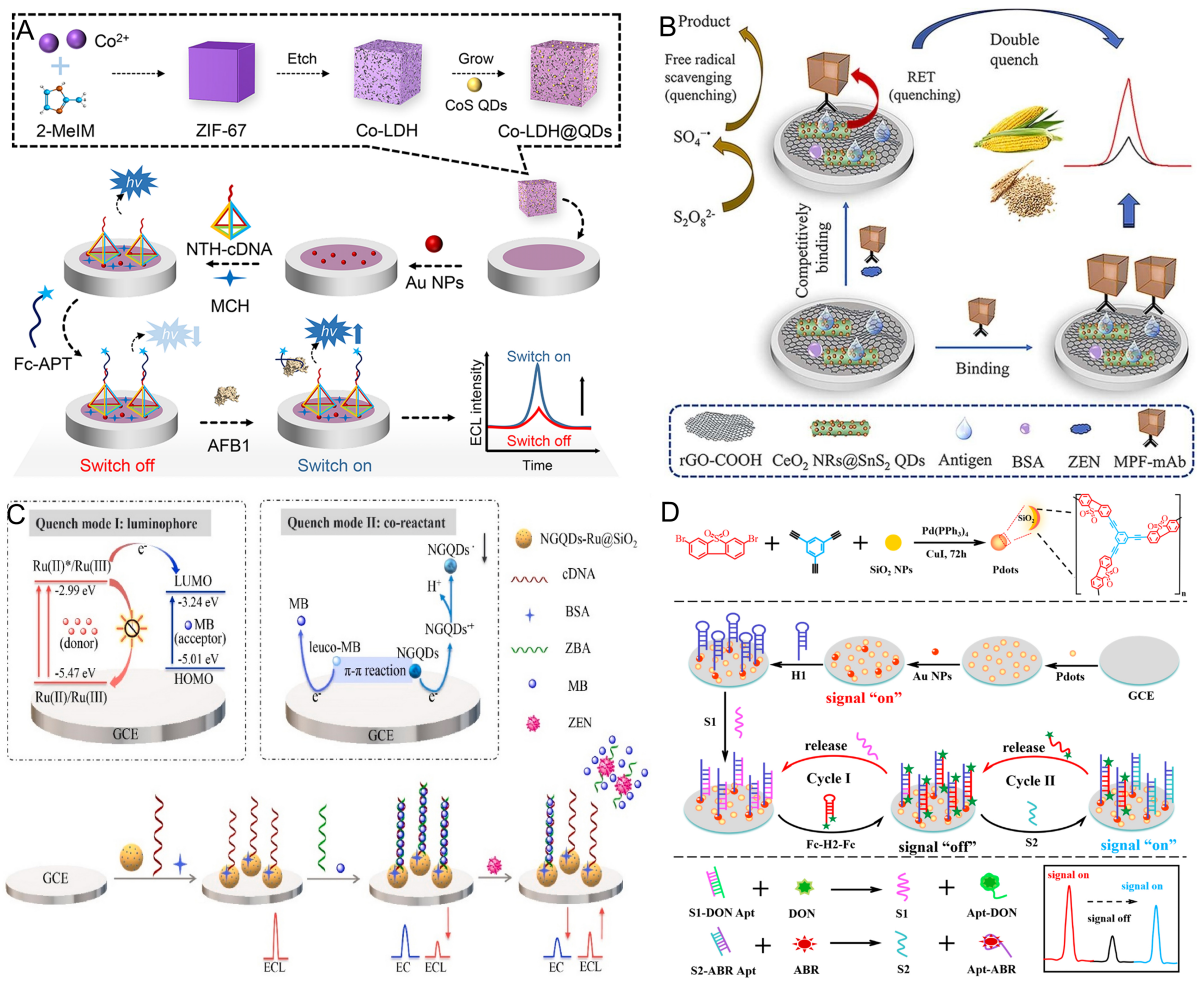
(**A**) Diagram of a switch-type ECL aptasensor combined with Co-LDH@QDs for AFB1 analysis. The blue and red curves represent the ECL responses with and without AFB1, respectively. Reprinted with permission from Ref. [76]. Copyright 2024, American Chemical Society. (**B**) Diagram of an ultrasensitive ECL immunosensor with a dual-mode quenching mechanism for detecting ZEN. The black and red curves represent the ECL responses with and without ZEN, respectively. Reprinted with permission from Ref. [79]. Copyright 2025, Elsevier. (**C**) Schematic diagram of the working mechanism of an innovative ratiometric ECL-EC aptasensor utilizing an MB-mediated dual-quenching effect for the sensitive detection of ZEN. The red and blue curves represent the ECL and EC intensity, respectively. The arrows represent the intensity variation trend. Reprinted with permission from Ref. [80]. Copyright 2022, Elsevier. (**D**) Illustration of a highly sensitive Pdot-based ECL biosensor for the sequential analysis of DON and abrin. The red, black and blue curves represent the ECL intensities without targets, with DON and abrin, respectively. Reprinted with permission from Ref. [81]. Copyright 2024, American Chemical Society.

The highly toxic and carcinogenic Aspergillus toxins, including AFB1 and ochratoxin A (OTA), are mainly metabolites synthesized by Aspergillus species, a fact that has raised worldwide concern due to their detrimental risks to human health [73,74]. Very recently, Chen’s group [75] manufactured an extremely sensitive ECL sensor armed with S-vacancy-modified MXene QDs anchored on a SnS_2_ nanoflower (MQD@SnS_2_) heterojunction as a new ECL luminophore for AFB1 analysis in dried fish samples. This heterojunction facilitates electronic transmission, suppresses electron–hole recombination, and boosts cross-interfacial charge transfer, which is beneficial for improving ECL efficiency. A quenched ECL intensity was observed when the target AFB1 was present, which was attributed to its targeted binding to the Apt-decorated MQD@SnS_2_ and AFB1 to cause the detachment of the composite from the electrode surface, eventually achieving a selective quantitative analysis of AFB1 in the detection range of 0.001–100 ppb. Hu et al. [76] developed a creative switch-type ECL aptasensor to quantitatively measure AFB1 by utilizing the encapsulation of cobalt–sulfur QDs into hollow cobalt-layered double hydroxide nanocages (Co-LDH@QDs) with an ECL nanoprobe and Fc-modified aptamer (Fc-Apt) as an ECL quencher. In this design, Co-LDH@QDs, Au NPs, and a cDNA-modified DNA nanotetrahedron (NTH-cDNA) were sequentially coated on the electrode, followed by subsequent hybridization with Fc-Apt, thereby resulting in effective ECL quenching via efficient electron transfer from Fc to Co-LDH@QDs without the target AFB1. In the presence of AFB1, the specific recognition between the given target and Fc-Apt enabled the separation of the complex from the electrode interface, deriving an enhanced ECL intensity dependent on AFB1 concentration. According to the ECL quenching mechanism, the proposed ECL sensing strategy had satisfactory analytical performance in determining AFB1, with a LOD as low as 0.03 pg/mL (Figure 3A). In addition, Gao et al. [77] proposed a sensitive ECL-RET sensing platform between CdTe QDs and Cy5 for OTA determination in a maize sample. The CdTe QDs, as the ECL donors, showed stable ECL emission and were conjugated with cDNA through a cross-linking system of chitosan and GA. Further hybridization reaction with Cy5-Apt led to a marked improvement in the ECL signal through the effective RET effect from QDs to Cy5. When OTA was added, the Cy5-Apt left the electrode surface because OTA interacted specifically with Apt, making for a substantial drop in the ECL signal. Considering the ECL signal variation, the designed ECL aptasensor enabled a highly sensitive and accurate quantification of OTA with a LOD of 0.17 pg/mL. Jia et al. [78] developed a sensitive CdSe@CdS QD-based ECL aptasensor for OTA detection. The successive modification of CdSe@CdS QDs, chitosan, and GA onto the electrode not only generated high ECL intensity but also provided a platform for the anchoring of the OTA aptamer to fabricate the sensing interface. The reduction in ECL signal induced by the highly specific recognition of the target, OTA, by its aptamer was used to quantify OTA in real Lily and Rhubarb samples, and had a wide detection range of 1–100 ng/mL with a LOD of 0.89 ng/mL.

Fusarium toxins (e.g., DON, ZEN, etc.) are primarily produced as toxic metabolites by Fusarium fungi in crops, which have adverse effects on the neurological, reproductive and immune systems of humans and animals [82,83]. Wang’s group [79] designed a novel ECL immunosensor integrated with the dual-quenching effect of a metal polydopamine framework (MPF) for the supersensitive detection of ZEN in corn and wheat. In this work, SnS_2_ QD-modified CeO_2_ nanorod (CeO_2_ NRs@SnS_2_ QDs) composites synergistically amplified ECL intensity, which was caused by the synergistic effect of CeO_2_ NRs as a co-reactant accelerator and the reduced carboxylated graphene oxide (rGO-COOH), which had good electrical conductivity. After covalently binding to ZEN antigens, the sensing system still maintained a stable and efficient ECL signal. The ECL signal was strongly quenched when MPF-mAb was added, attributed to both a potent radical scavenger and the ECL receptor of MPF. Upon adding the target ZEN, a weakened ECL signal was detected, which was explained by the competition for binding on the MPF-mAb specific sites between ZEN and the immobilized antigens. The developed ECL sensor demonstrated a wide linear range, spanning from 0.001 to 500 ng/mL for ZEN quantification, with a LOD of 0.103 pg/mL (Figure 3B). Meanwhile, Luo et al. [80] reported an ECL-EC ratiometric aptasensor based on NGQDs-Ru@SiO_2_ as the ECL probe and methylene blue (MB) as the quencher for sensitive and accurate analysis of ZEN. The significant ECL intensity of NGQDs-Ru@SiO_2_ arises from the intramolecular reaction between NGQDs and Ru@SiO_2_. Following this, cDNA was bound with the ZEN aptamer to form a double-stranded DNA (dsDNA) structure that enabled the absorption of MB, yielding a satisfactory EC signal. Concurrently, a substantial reduction was observed in the ECL signal of NGQDs-Ru@SiO_2_, primarily driven by two ECL quenching mechanisms of the ECL-RET effect, electron transfer, and π-π conjugation between NGQDs-Ru@SiO_2_ and MB. Upon the specific interaction of the target ZEN and its aptamer, the aptamer and MB were displaced from the sensing surface, producing a recovery of the ECL signal and a concurrent decrease in EC signal. Therefore, this proposed ratiometric sensing strategy offers an ultrasensitive and precise approach for quantifying ZEN, coupled with a remarkably low LOD of 0.85 fg/mL (Figure 3C). In recent years, multi-target detection within the domain of ECL methods has attracted considerable research attention. Xiang et al. [81] proposed an ultrasensitive “on–off–on” ECL aptasensing platform combined with AIE-active Pdots for the sequential and quantitative measurement of DON and abrin (ABR). As seen in Figure 3D, the sequential modification of Pdots and AuNPs displayed high ECL intensity as a “signal on” state. The specific binding of target DON to its aptamer upon administration induced a strand displacement reaction to shed S1 from the S1-Apt hybrid formed by hybridization of S1 and the DON aptamer. The liberated S1, as a catalytic trigger, started the follow-up cycle I to open the hairpin H1 and allowed for the capture of hairpin Fc-H2-Fc, which greatly attenuated ECL intensity in the “signal off” state because of the considerable quenching effect caused by the occurrence of ECL-RET between Pdots and Fc. Following the addition of ABR, it specifically bound with the aptamer to liberate S2 as the cycle II for replacing hairpin Fc-H2-Fc, thus detaching Fc-H2-Fc from the sensing interface and enhancing ECL intensity in the “signal on” state. Given these two cycles, the ECL aptasensing platform for the simultaneous determination of DON and ABR exhibited a lower LOD of 0.73 fg/mL and 0.38 pg/mL, respectively.

### 3.3. Detection of Antibiotics

Antibiotics, which function by inhibiting and eliminating bacterial growth, have found widespread use across healthcare, livestock farming, and agriculture, contributing significantly to societal economic progress [84,85]. They act as typical feed additives in animal husbandry for disease prevention and growth promotion, but excessive use in animal-derived products could produce deleterious residues that harm human health upon ingestion via the food chain [86]. Accordingly, ECL sensors based on nanoaggregates have been widely developed for antibiotic determination.

**Figure 4:**
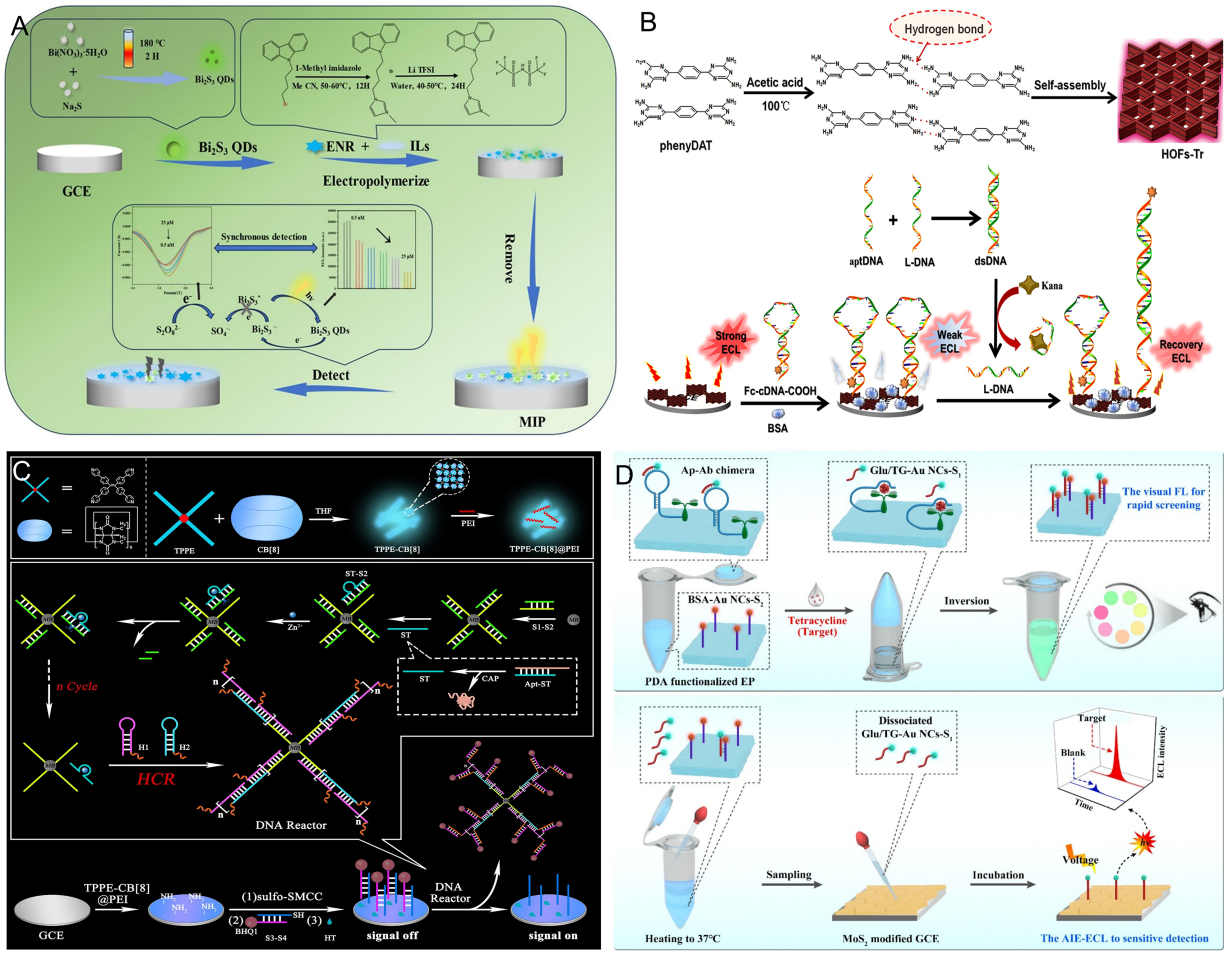
(**A**) Schematic diagram of a novel ECL/DPV dual-mode sensor based on Bi_2_S_3_ QDs for ENR assay in eggs. Reprinted with permission from Ref. [88]. Copyright 2024, Elsevier. (**B**) Diagram of a Tr-HOFs-based ECL aptasensing strategy for the sensitive measurement of KAN. Reprinted with permission from Ref. [91]. Copyright 2021, American Chemical Society. (**C**) Diagram of an ultrasensitive ECL sensor with AIE-active CB[8]-TPPE for detecting CAP. Reprinted with permission from Ref. [92]. Copyright 2024, Elsevier. (**D**) Illustration of a portable FL-ECL dual-signal sensor coupled with Glu/TG-Au NCs for detecting TC. The red and blue curves represent the ECL responses in the presence and absence of TC, respectively. Reprinted with permission from Ref. [93]. Copyright 2025, Elsevier.

The irrational use of ENR, a pioneering veterinary fluoroquinolone valued for its potent bactericidal efficacy, wide antibacterial spectrum, and excellent safety profile, has inadvertently led to residue being found in animal products and environmental pollution, bringing about various side effects on human health, such as antibiotic resistance, gut microbiota imbalance, and allergic reactions [87]. In this instance, Liu et al. [88] engineered an innovative dual-mode MIP sensor based on bismuth sulfide QDs (Bi_2_S_3_ QDs) as the ECL tag for synchronous differential pulse voltammetry (DPV) and ECL detection of ENR in eggs. A prepared MIP film composed of Bi_2_S_3_ QDs, ILs, and the template ENR was electropolymerized on the electrode to form numerous imprinted cavities. The subsequent recognition and capture of ENR by these cavities hindered the electron transfer at the electrochemical interface and caused a consequent suppression of both the DPV and ECL responses. This synchronous ECL and DPV dual-mode sensor for the precise measurement of ENR had a LOD of 0.13 nM and 1.59 nM, respectively (Figure 4A). Zhang et al. [89] fabricated a sensitive ECL sensing platform with the ligand-regulated terbium ions-doped ovalbumen (OVA)-protected CuNCs (Tb-OVA-Cu NCs) for the trace detection of ENR. By utilizing OVA as a stabilizer to incorporate Tb^3+^ ions into the framework of the sensor, the Tb-OVA-Cu NCs with intense anodic ECL emission were prepared for further modifying aptamer2. A substantial change in ECL intensity was observed with ENR to aptamer1 and Tb-OVA-Cu NCs-aptamer2, indicating that this sensing platform enabled the selective determination of ENR with an LOD as low as 0.06 pg/mL. Also, Chen’s group [90] constructed a highly sensitive near-infrared ECL (NIR-ECL) aptasensing platform combined with AgBr NC-decorated Ti_3_C_2_ MXene (Ti_3_C_2_-AgBr NCs) composites for ENR analysis by specifically combining it with its corresponding aptamer. A strong NIR-ECL emission from the Ti_3_C_2_-AgBr NCs was generated by the surface defect effect of O-terminated Ti_3_C_2_ MXene. The decoration of the ENR aptamer onto the resulting Ti_3_C_2_-AgBr NCs sharply weakened the ECL signal because of the hindrance of electron transfer by its aptamer. Upon introduction of the target ENR, the ECL signal was regenerated as a result of the dissociation of the ENR–aptamer complex, facilitating ultrasensitive ENR quantification with a remarkable LOD of 0.597 pM.

As a broad-spectrum antibiotic, KAN is commonly employed as a growth-promoting feed additive in animal husbandry, the excessive use of which may increase antimicrobial resistance and disrupt human gut microflora, directly affecting consumers’ health [94,95]. Zhang et al. [91] constructed a label-free ECL aptasensor based on cuboid-like Tr-HOFs as a promising ECL emitter and cDNA-Fc as the quencher for KAN determination. The resulting Tr-HOFs were synthesized through the N···H hydrogen bond-driven self-assembly of 6,6′-(1,4-phenylene)bis(1,3,5-triazine-2,4-diamine), which had robust ECL emission with a high ECL efficiency of 21.3%. The ECL signal was efficiently quenched by assembling cDNA-Fc on the Tr-HOFs-modified electrode. In contrast, the subsequent addition of KAN restored the ECL intensity because KAN’s specific recognition of the aptamer caused the competitive release of L-DNA from dsDNA and displaced the cDNA-Fc quencher, ultimately achieving highly selective detection of KAN with an LOD of 0.28 nM (Figure 4B). Ouyang’s group [96] developed an ultralow potential ECL aptasensor armed with DNA nanoribbon template self-assembly CuNCs (DNR-CuNCs) as a coreaction accelerator for sensitive detection of KAN in milk. The designed DNR-CuNCs catalyzed H_2_O_2_ reduction to preferentially yield potent hydroxyl radical species, effectively amplifying cathodic ECL intensity in the luminol-H_2_O_2_ system. The strong π-π stacking interactions between the KAN aptamer and graphene worked as an effective signal on/off switch. Upon KAN addition, the competitive affinity of KAN for its aptamer triggered the displacement of the DNR-CuNCs from the electrode to inhibit the generation of ECL signal in the luminol-H_2_O_2_ system, allowing it to quantitatively detect KAN with a LOD as low as 0.18 fg/mL. Beyond this, Feng et al. [97] reported a novel di-gears ECL aptasensor incorporated MIL-53(Fe)@CdS composites for the simultaneous detection of KAN and neomycin. In this study, the sensing system exhibited a weak ECL signal with dual gears in the “OFF” state without targets as a result of an effective ECL-RET process. In contrast to the initial “OFF” state, the KAN bound with its aptamer to turn the gears “ON” and increase the ECL signal via the SPR effect of CdS QDs and AuNPs. On the contrary, the subsequent binding of the neomycin aptamer returned the sensing system to the “OFF” state, decreasing the ECL signal by activating ECL-RET among them. This “OFF” state was reversed by adding neomycin that fully hybridized with its aptamer, removing it from the electrode and turning the gears “ON” state to restore ECL through the re-established SPR effect. Based on its unique design, this aptasensor allowed for the multiplexed ECL detection of both KAN and neomycin with a relatively low LOD of 17 pM and 0.35 nM, respectively.

Chloramphenicol (CAP) is a low-cost and broad-spectrum antibiotic effective against diverse pathogens and has broad applications in animal husbandry, but its overuse imposes many side effects on the environment and human health, encompassing gastrointestinal disturbances, bone marrow regeneration, and neurological disorders [98]. For this reason, Li and his colleagues [99] proposed a competitive ECL-RET immunosensing system between SnS_2_ QDs and Ag@Au NSs for ACP determination originating from the specific response between antigen and antibody. Among them, the flower-like ZnO NFs as the nanocarrier could load more SnS_2_ QDs and coat antigens to gain a satisfactory ECL signal. The antibody-decorated Ag@Au NSs were conjugated with ZnO NFs@SnS_2_ QDs through the specific antigen–antibody interaction to cause an ECL-RET process to occur to further lower ECL intensity. Conversely, the presence of CAP competed with the coating antigen for limited antibody binding sites in the competitive immunoreaction, thereby reducing the attachment of excess Ag@Au NSs to obtain higher ECL intensity. As a consequence, this proposed ECL immunosensor was capable of measuring ACP with a wide detection range of 0.005–1000 ng/mL and a lower LOD of 1.7 pg/mL. Chen et al. [92] designed a new ratiometric ECL sensor based on the supramolecular assembly of cucurbit[8]uril and 1,1,2,2-tetrakis(4-(pyridin-4-yl) phenyl)-ethene (TPPE) (CB[8]-TPPE) complex with an excellent dual-AIECL behavior for the ultrasensitive determination of CAP in honey and milk as result of the highly specific response to its aptamer. In this work, the CB[8]-TPPE was fixed on the electrode surface and bonded with MB/S1-S2, efficiently degrading dual-AIECL signals due to the quenching behavior of BHQ1 towards TPPE. The introduction of CAP activated a DNA reactor through Zn^2+^-specific cleavage binding HCR that further coupled with BHQ1-labeled S4, causing it to detach from the electrode, and restore the dual AIECL signals for a sensitive CAP assay with the LOD down to 1.81 fg/mL (Figure 4C).

Tetracycline (TC) serves as a commonly used broad-spectrum antibiotic in clinical practice that has favorable inhibitory effects on various bacteria to effectively control animal infectious diseases [100]. Nevertheless, its abuse led to residual accumulation in animal-derived foods, thereby threatening ecological stability and public safety. Yi et al. [101] reported a novel AIE-active OVA-confined tetrakis(4-aminophenyl)ethene (OVA/ETTA)-based ECL sensing platform for detecting TC in milk due to the target-aptamer-specific binding. The prepared OVA/ETTA composite with abundant nanocavities and active sites allowed for both ECL signal stabilization and efficient aptamer conjugation. The use of the co-reaction accelerator NH_2_-Fe-MOF substantially boosted the ECL response by generating more free radicals, which enabled the accurate quantification of TC from 0.1 pM to 1 uM with a low LOD of 42.6 fM. Ma’s group [93] developed a portable point-of-care testing platform by using a dual-color Au NC probe with efficient FL and ECL dual-emissions for on-site rapid monitoring of TC in Eppendorf (EP) tubes. The target TC recognition by an aptamer–antibody (Ap-Ab) chimera produced visual PL signals via green-emitting Glu/TG-Au NCs-S1 and red-emitting BSA-Au NCs-S2 for on-site rapid screening of high concentration positive samples. The weakly bonded S1-S2 duplex dissociated upon heating to release S1 to trigger a highly sensitive ECL testing process. The dual-signal sensor showed a wide dynamic range of 5 fM-1 uM, accompanied by a LOD of 73 fM for visual FL readout and 2.3 fM for ECL readout, respectively (Figure 4D).

### 3.4. Detection of Pathogens

The widespread distribution of pathogens in the environment enables them to contaminate the entire food chain from raw materials to processed products. If consumers accidentally consume food infected with pathogens, they can cause diverse diseases [102,103,104]. Consequently, food contamination caused by foodborne pathogens has escalated into a global public health issue, constituting a major hazard to human health and safety. Despite various existing methods for detecting foodborne pathogens in foods, the limitations of false positive results or dummy signals remain a major challenge [105]. Thus, ECL sensors based on nanoaggregates were developed to address these limitations, which was very important for the rapid detection of pathogens to reduce human health risks.

**Figure 5:**
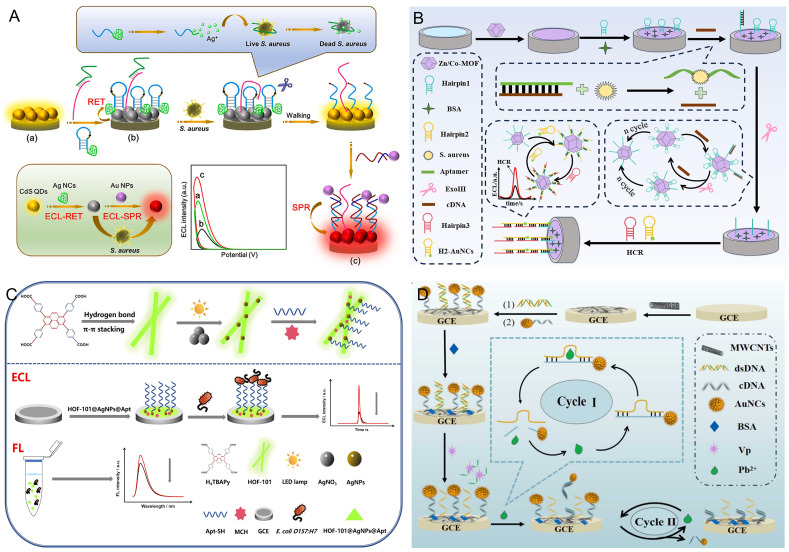
(**A**) Schematic illustration of a stimulus-responsive ECL sensor for detecting and sterilizing *S. aureus* in foods. Reprinted with permission from Ref. [107]. Copyright 2022, Elsevier. (**B**) Schematic diagram of a sensitive ECL biosensor based on Arg/ATT-Au NCs for *S. aureus* sensing. Reprinted with permission from Ref. [108]. Copyright 2025, Elsevier. (**C**) Diagram of a novel ECL/FL aptasensor with HOF-101@AgNPs for detecting *E. coli O157:H7*. The arrows represent the trend of the intensity changes with and without *E. coli O157:H7*, respectively. Reprinted with permission from Ref. [110]. Copyright 2024, Elsevier. (**D**) Illustration of a highly sensitive Met-AuNC-based ECL sensor for *VP* determination. Reprinted with permission from Ref. [112]. Copyright 2024, Elsevier.

*Staphylococcus aureus* (*S. aureus*) is a ubiquitous Gram-positive bacterium, prone to causing sepsis, toxic shock syndrome, and enteric infections [106]. Based on this, Feng and her colleagues [107] constructed a stimulus-responsive ECL sensing system based on a DNA walker and the combined mechanisms of RET and SPR for the sensitive detection and in situ sterilization of *S. aureus.* By employing AgNC-functionalized hairpin DNA (H-AgNCs) as the energy acceptor, this sensor achieved significant quenching of CdS QDs’ ECL emission as the energy donor through the ECL-RET mechanism. The presence of *S. aureus* triggered DNA walking and nicked endonuclease cleavage to liberate H-AgNCs from the QD surface. Followed by the introduction of AuNPs, ECL signal enhancement was realized by an energy transfer from the SPR of Au NPs to CdS QDs. Meanwhile, the released Ag+ ions from H-AgNCs achieved the instantaneous killing of bacteria during the detection process by destroying cell membranes and disturbing DNA/RNA replication. Based on the above mechanisms, this developed ECL sensor was capable of both quantitatively detecting *S. aureus* in foods, with a LOD as low as 1.0 CFU/mL, and simultaneously sterilizing them (Figure 5A). Similarly, Zhang’s group [108] developed a novel Arg/ATT-Au NCs-based ECL sensor combined with a cascade signal amplification strategy of an enzyme-catalyzed DNA walker and HCR for low-abundance *S. aureus* determination in seafoods involving scallops, fish, and shrimp. A signal enhancement in the ECL intensity of Arg/ATT-Au NCs was observed by the host–guest structures. In the presence of *S. aureus*, it specifically reacted with its aptamer to liberate the walker chain, which further triggered the ExoIII-mediated DNA walker of hairpin1 (H1) on the Zn/Co-MOF surface. Subsequently, massive digested fragments reacted with AuNCs-labeled H2 and H3 to improve the conversion efficiency of H1 into HCR products, causing a marked surge in the ECL intensity, which showed linearity against the logarithm of *S. aureus* concentration over the range of 10–109 CFU/mL, and its LOD was estimated at 1.16 CFU/mL (Figure 5B).

As one of the most highly dangerous Gram-negative bacteria, *Escherichia coli O157:H7* (*E. coli O157:H7*) infects humans through contaminated food, water, or direct contact, causing a series of diseases, from abdominal pain and diarrhea to hemorrhagic colitis, and potentially fatal complications like acute renal failure, which constitutes a grave public health threat because of its high mortality and associated permanent sequelae [109]. For this reason, Yang et al. [110] proposed an innovative ECL/FL dual-mode sensor for ultrasensitive determination of *E. coli O157:H7* using HOF-101@AgNPs as a new-type ECL probe. HOF-101 with outstanding ECL and FL characteristics was prepared by the hydrogen bonding and π-π stacking interaction of 1,3,6,8-tetra (4-carboxyphenyl) pyrene (H_4_TBAPy). The HOF-101@AgNPs@Apt composites were then synthesized by the in situ photoreduction of HOF-101 and AgNO_3_ for amplifying the ECL intensity of HOF-101 as well as the further immobilization of Apt with Ag-S bonding for identifying targets. After adding *E. coli O157:H7*, both the ECL and FL intensity of the HOF-101@AgNPs@Apt composites were dramatically decreased, resulting from the impeded electron transport of Apt and the photo-induced electron transfer, eventually achieving high-precision ECL and FL detection of *E. coli O157:H7* with LODs of 0.48 CFU/mL and 2.39 CFU/mL, respectively (Figure 5C). As another typical Gram-negative bacterium, *Vibrio parahaemolyticus* (*VP*) is regarded as a primary pathogen responsible for foodborne gastrointestinal illness in humans [111]. Yan et al. [112] developed a methionine-capped AuNC (Met-AuNC)-based ECL sensing platform coupled with a cascade signal amplification strategy for highly sensitive detection of *VP* in scallops. A dsDNA hybrid of the *VP* aptamer, a DNA walker, and AuNC-labeled cDNA were fixed to the electrode surface in turn. The introduction of *VP* competitively interacted with its aptamer, leading to the dissociation of dsDNA and the exposure of the DNAzyme fragment in the DNA walker. Further introducing Pb^2+^ activated the DNAzyme-powered DNA walker, initiating its circulation and cDNA cleavage on the electrode surface to attenuate the ECL intensity of Met-AuNCs. As a result, the developed ECL sensor for the *VP* assay exhibited an LOD as low as 1.23 CFU/mL (Figure 5D).

### 3.5. Detection of Heavy Metal Ions

In the recent past, food safety issues arising from heavy metal pollution have become increasingly serious. These heavy metals can accumulate in organisms over time through the food chain, causing chronic poisoning and irreversible damage to human health [113,114]. Hence, monitoring and controlling heavy metals in food is essential for safeguarding food security. Nowadays, traditional detection approaches relying on large-scale equipment fail to meet the requirements for on-site testing of heavy metals. Benefiting from their high sensitivity, convenient operation, and low cost, nanoaggregate-based ECL sensors are an ideal solution for the rapid detection of trace heavy metal ions, holding considerable promise in the food safety field.

**Figure 6:**
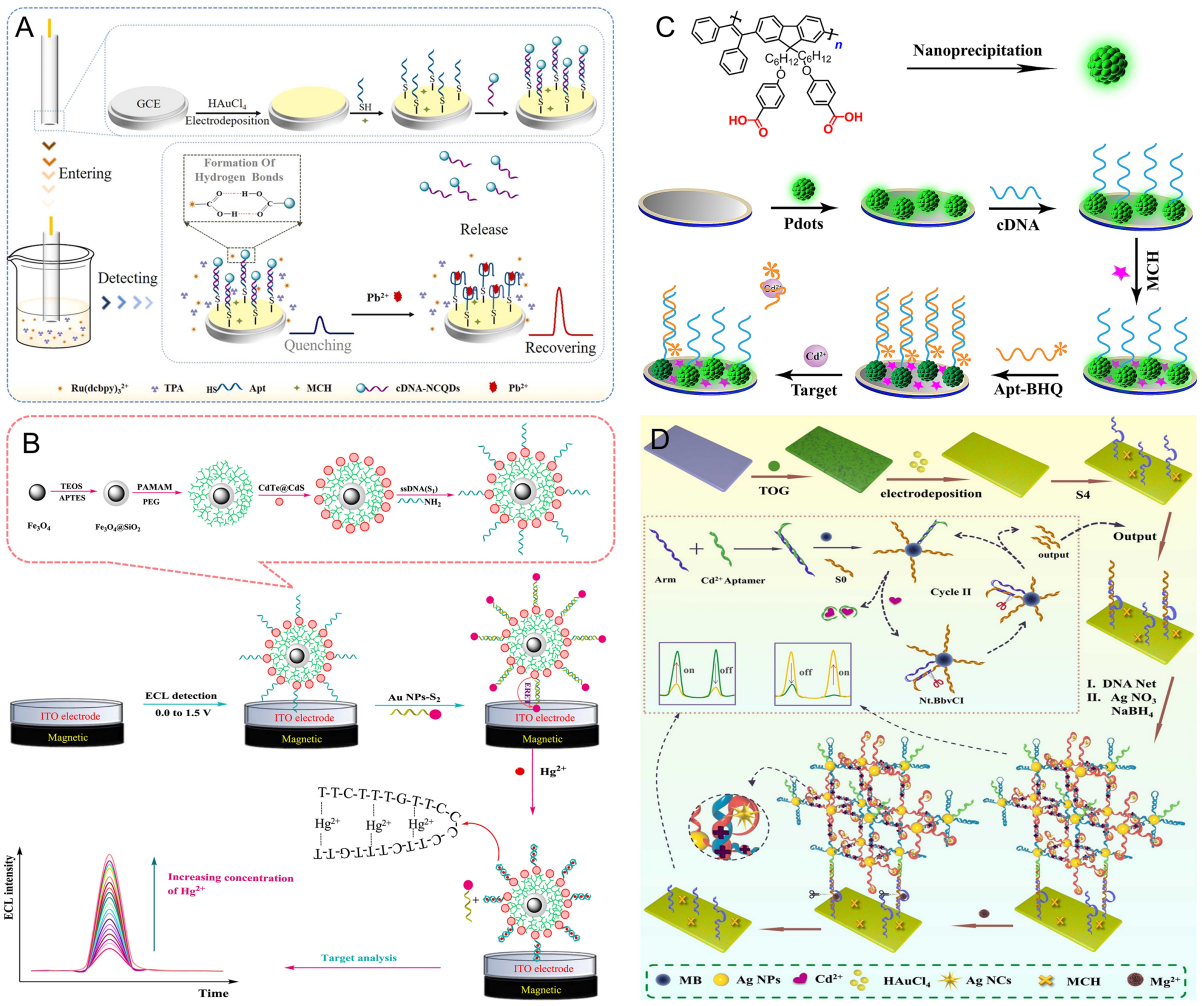
(**A**) Schematic illustration of an ultrasensitive “off–on” ECL biosensor for detecting Pb^2+^ with a dual signal amplification strategy. Reprinted with permission from Ref. [117]. Copyright 2021, Elsevier. (**B**) Illustration of a new switchable ECL-RET aptasensing platform for Hg^2+^ measurement in environmental and fishery samples. The colors represent the ECL responses of different concentration of Hg^2+^. Reprinted with permission from Ref. [118]. Copyright 2018, Elsevier. (**C**) Diagram of a highly sensitive ECL-RET aptasensor based on AIE-active Pdots for Cd^2+^ detection in Ganoderma lucidum. Reprinted with permission from Ref. [119]. Copyright 2024, Elsevier. (**D**) Diagram of a dual-potential ECL biosensor with DNA Net probes for multiplexed determination of Cd^2+^ and Mg^2+^. Reprinted with permission from Ref. [120]. Copyright 2022, Elsevier.

As a toxic heavy metal, lead ion (Pb^2+^) tends to bioaccumulate in vivo, having detrimental influences on the nervous and respiratory systems [115]. You’s group [116] developed a sensitive “on–off–on” ECL biosensor using a dual-amplification strategy with both the AIE and RET effects for monitoring Pb^2+^ in soil as a consequence of the specific binding between the target and its aptamer. First of all, an intense and stable ECL signal of AIE-active AuNCs was obtained (signal “on” state). The rhodamine B (RhB), as the energy acceptor, was encased in a double-stranded hybrid of cDNA and Apt to quench the ECL signal of the AuNCs (signal “off” state) when it detected the occurrence of RET. After the introduction of Pb^2+^, its specific recognition to Apt resulted in the escape of RhB from the sensing interface, thereby blocking the RET process between them and recovering the ECL signal of the NCs (signal “on” state). In view of the dual-signal amplification strategy, the developed sensor achieved highly sensitive detection of Pb^2+^ in soil samples with a LOD of 23 pM. Soon afterwards, this group [117] reported a novel “off–on” ECL aptasensing platform with the synergistic signal amplification strategy of the detachment of quencher NCQDs and generation of G-quadruplex for supersensitive determination of Pb^2+^ in soil and water. In this approach, based on the specific hybridization between the Pb^2+^ aptamer and cDNA, cDNA-NCQDs were assembled at the electrode interface and served as a quencher of the tris(4,4′-dicarboxylic acid-2,2′-bipyridyl)ruthenium(II)/tripropylamine (Ru(dcbpy)_3_^2+^/TPA) system to decrease its ECL emission through an energy-transfer-quenching mechanism induced by the intermolecular hydrogen bonds. The presence of the target Pb^2+^ triggered a specific binding event with its aptamer, leading to the abscission of cDNA-NCQDs from the electrode surface as well as the simultaneous formation of G-quadruplex, both of which contributed to a greatly increased ECL intensity. This synergistic amplification effect endowed the ECL aptasensor with high sensitivity for Pb^2+^ detection, demonstrating a wide detection range from 1 fM to 10 nM and an ultra-low LOD of 0.19 fM (Figure 6A).

Mercury ion (Hg^2+^) also presents a non-ignorable endanger to public health, underscoring the need for reliable detection strategies [121]. Given this, Babamiri et al. [118] proposed an “on–off–on” switchable ECL-RET aptasensing strategy for the quantitative detection of Hg^2+^ based on Fe_3_O_4_@SiO_2_/dendrimers/CdTe@CdS QD nanocomposites as ECL nanoemitters and AuNPs as quenchers. In their work, they designed nanocomposites that first generated a strong ECL intensity (signal “on” state). When they covalently bound with aminated T-rich single-stranded DNA (S1) and further hybridized with cDNA grafted AuNPs (AuNPs-S2), their ECL intensity was significantly reduced (signal “off” state) due to the valid RET of AuNPs and QDs. Upon the introduction of Hg^2+^, they formed a strong and stable T-Hg^2+^-T complex with S1, accompanied by the liberation of AuNPs-S2 in the nanocomposites, thereby recovering the QDs’ ECL intensity (signal ‘‘on” state). On the basis of this mechanism, the ECL-RET aptasensor achieved the attomolar-level determination of Hg^2+^ in tap water, carp, and saltwater fish, with a recorded LOD as low as 2 aM (Figure 6B). Afterward, Hua et al. [122] developed an ultrasensitive label-free ECL sensing platform armed with europium sulfide nanocrystals (EuS NCs) for an Hg^2+^ assay in seafood. The EuS NCs generated a strong cathodic ECL signal with K_2_S_2_O_8_ as the co-reactant. Upon addition of Hg^2+^, a marked reduction in the ECL intensity was recorded thanks to the formation of Hg-S bonds between Hg^2+^ and S^2-^ from the EuS NCs, which effectively suppressed ECL emission. Taking advantage of this quenching mechanism, the proposed ECL sensor achieved an impressive LOD of 0.028 pM, enabling the successful monitoring of trace Hg^2+^ in real seafood samples like fish, shrimp, and conch.

In addition to this, cadmium ions (Cd^2+^) with high toxicity and carcinogenicity can cause various diseases involving cardiopathy and diabetes, along with damage to vital organs like the liver and kidneys [123,124]. Xu et al. [119] engineered a supersensitive ECL-RET sensor based on AIE-active Pdots and BHQ as the energy donor–acceptor pairs for Cd^2+^ measurement in Ganoderma lucidum. Among them, the AIE-active Pdots prepared by the nano-coprecipitation method were decorated on the GCE surface and covalently conjugated with cDNA. When specifically hybridized with BHQ-labeled aptamer (Apt-BHQ) via complementary base pairing, a pronounced quenching of ECL intensity was observed as a result of the RET process between BHQs and the Pdots. The recovered ECL signal was obtained with the addition of Cd^2+^, which resulted in the detachment of Apt-BHQ from Pdots through the specific binding between Cd^2+^ and its aptamer. This principle gave the sensor an excellent capability to detect Cd^2+^, achieving a LOD as low as 0.006 ppb (Figure 6C). Moreover, the simultaneous detection of multiple metal ions is an emerging paradigm in the development of ECL sensors. Lately, Jie’s group [120] reported a versatile dual-potential ECL aptasensor for simultaneous detection of Cd^2+^ and Mg^2+^ based on terbium organic gel (TOG) and Ru(phen)_3_^2+^ as dual-ECL emitters and AgNCs as quenchers. In this design, the DNA network (DNA Net) with C-rich bases was first assembled by multiple cycles of S1-H1 hybridization and used as the carrier to load a large amount of Ru(phen)_3_^2+^ (DNA Net@Ru) to obtain a strong positive-potential ECL. In the existence of Cd^2+^, the DNA Net@Ru was anchored to the TOG-modified electrode via abundant output DNA generated by the DNA walker amplification strategy, which greatly enhanced the ECL response of Ru(phen)_3_^2+^ to Cd^2+^, and facilitated the in situ formation of numerous AgNCs on C-rich bases to quench the ECL response of TOG to Cd^2+^. The subsequent introduction of the target, Mg^2+^, precisely executed the cleavage of the targeted DNA enzymes to cause the detachment of DNA Net with Ru(phen)_3_^2+^ and AgNCs from the electrode, thereby diminishing the ECL signal of Ru(phen)_3_^2+,^ and which in turn restored the ECL signal of TOG (Figure 6D). Among the different ions present, only Cd^2+^ and Mg^2+^ caused great changes in the ECL signals of the sensor. In view of this, the multifunctional dual-potential ECL sensing platform for monitoring Cd^2+^ in rice strongly demonstrated its great potential for practical multi-target analysis.

### 3.6. Detection of Illegal Additives

**Figure 7:**
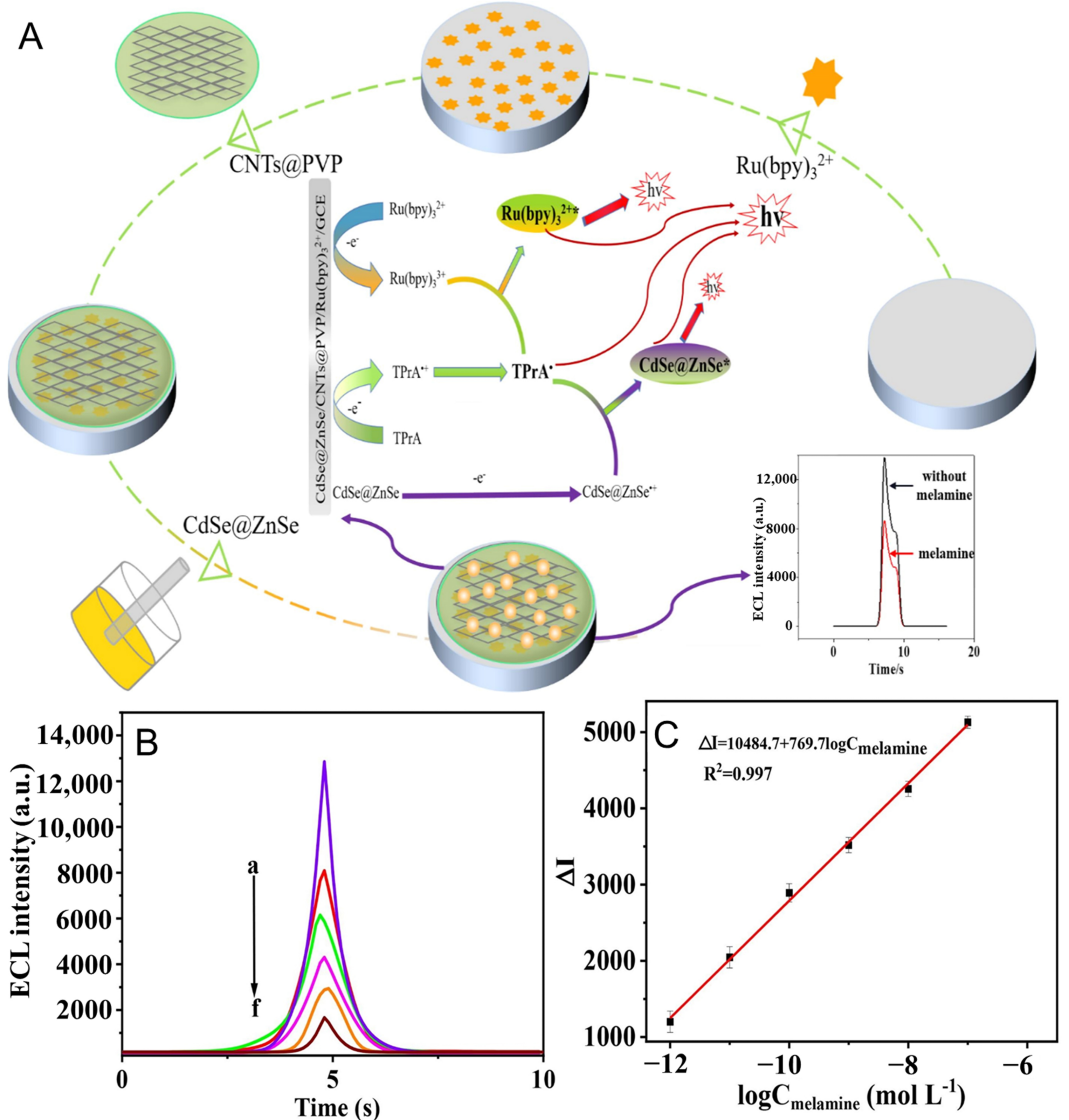
(**A**) Illustration of a highly sensitive ECL sensor for melamine assays in milk. (**B**) ECL responses of the sensor for detecting different concentrations of melamine. The ECL curves in (a) without and (f) with 0.1 uM melamine, respectively; The arrow represents the trend of melamine concentration change. The different color curves represent the ECL responses in different concentrations of melamine, respectively. and (**C**) its corresponding linear calibration curve. Reprinted with permission from Ref. [127]. Copyright 2023, Elsevier.

Technological advancements fuel the development of the food industry, but also bring latent hazards. Driven by the pursuit of profit, some businesses have widely added cheap and illegal additives like melamine and Sudan dyes, seriously endangering consumers’ health and safety. In this context, many analytical approaches based on chromatography, mass spectrometry, spectroscopy, and electrochemistry for the quantitative detection of these illegal additives in food products have been reported [125]. In comparison to other methods, ECL methods based on nanoaggregates offer the advantages of high sensitivity, zero background, and low cost, highlighting great promise for illegal additive detection in foodstuffs [126]. For example, Wu et al. [127] fabricated a sensitive ECL sensing strategy using CNTs as a carrier, Ru(bpy)_3_^2+^ as an ECL luminophore, and CdSe@ZnSe QDs as a co-reaction accelerator, for the highly selective monitoring of melamine in milk samples. As illustrated in Figure 7A, an amplified ECL intensity of the Ru(bpy)_3_^2+^/TPA system was detected when Ru(bpy)_3_^2+^, CNTs, and CdSe@ZnSe QDs were successively immobilized to the electrode, attributed to the collaborative effect of CNTs and QDs. With the increase in melamine concentration, their ECL intensities diminished significantly (Figure 7B). There was a good linear relationship between the relative ECL intensity and the logarithm of melamine concentration in the range of 0.001–100 nM with a LOD of 3.3 pM (Figure 7C). This designed ECL sensor proved effective for determining melamine in milk samples. In addition, the indiscriminate use of Sudan I as a food coloring agent may bring about significant risks to humans, such as carcinogenesis, mutagenicity, and the induction of pigmentary contact dermatitis. Thus, it is necessary to establish a rapid and effective method for detecting Sudan I in foods. Wang et al. [128] constructed a competitive ECL immunosensor combined with CdSe@CdS QDs as the signal probe, palladium/aurum core–shell nanocrystallines (Pd/Au CSNs), and GNR-functionalized graphene oxide (GNRs/GO) as the nanocarriers for ultrasensitive determination of Sudan I in tomato sauce, chili sauce, and chili powder because of the high-specificity binding of the antibody to the antigen. Pd/Au CSNs with large surface areas and superior catalysis could immobilize abundant QDs to prominently intensify ECL intensity. In parallel, GNRs/GO with good electrical conductivity functioned as an efficient substrate to load more coating antigens and promote electron transfer, which resulted in a synergistic amplification of the ECL signal. According to this design, a highly sensitive ECL immunoassay was developed for Sudan I, reaching an excellent LOD of 0.3 pg/mL, along with a broad linear dynamic range.

**Table 1:** Summary of nanoaggregate-based ECL sensors for detecting foodborne contaminants.

Nanoaggregates	Targets	Linear Ranges	LOD	Real Samples	Ref.
Ga@CQDs	cypermethrin	0.05–100 uM	0.03 uM	seawater, aquatic products	[15]
Au@AgNPs	profenofos	10^−4^–0.001 ng/mL	5.32 fg/mL	rape, spinach, cabbage	[23]
AgNPs	KAN	0.5–100 ng/mL	0.06 n/mL	milk	[25]
AuNPs	DON	10^−4^–10^2^ ng/mL	0.036 pg/mL	wheat, oat, rice, corn	[26]
CNGH	KAN	1 pM–50 nM	0.33 pM	milk	[28]
BNQDs	diazinon	3 fM–6.5 nM	0.95 fM	tap water, river water, apple, peach	[29]
M-Ag@MoS_2_-QDs	TBZ	0.5 nM–0.5 uM	0.142 nM	orange, potato, grape	[30]
Pdots	As(III)	10 pM–500 nM	5.8 pM	rice	[32]
PFN NPs	OPs	1 pM–0.5 uM	0.33 pM	pakchoi, cabbage, lettuce	[33]
AuNCs@CS nanogels	AFB1	0.0316–3.16 pg/mL	9.3 fg/mL	leech	[36]
CuNCs	OA	0.05–70 ng/mL	1.972 ng/mL	oyster	[37]
DPA CNPs	AFB1	10^−5^–100 ng/mL	3 fg/mL	fresh walnut	[39]
TPE NAs	ZEN	10^−6^–100 ng/mL	0.362 fg/mL	corn, wheat flour	[40]
HOF-101	OTC	0.1 pM–100 nM	0.04 pM	milk	[42]
CdTe QDs	CLB	0.6 nM–03 pM	0.1 pM	pork	[43]
AuNCs	KAN	5 × 10^−5^–50 ng/mL	32.9 fg/mL	milk, honey	[44]
CuNCs	ENR	0.1 nM–1 uM	27 pM	Beef, pork, pork liver, pomfret, bovine serum, human urine, lake water	[45]
CdS@ZnS QDs	permethrin	10 fM–100 nM	3.3 fM	celery, cabbage, spinach	[46]
Anth-CQDs	PAT	10^−4^–10 ng/mL	0.05 pg/mL	apple, apple juice, puree, fruit vinegar, fruit wine	[47]
CdS QDs	OPs	5.0 pM–0.5 nM	1.67 pM	pakchoi, cabbage, lettuce	[48]
BPPDs	PAT	5 × 10^−6^–0.5 ng/mL (ECL), 0.0001–1 ng/mL (FL), 0.0005–5 ng/mL (PT)	0.25 fg/mL (ECL), 34 fg/mL (FL), 0.2 pg/mL (PT)	Apple, hawthorn, peach, puree, Jam, apple juice	[50]
AgNPs	atrazine	0.001–1000 ng/mL	0.33 pg/mL	tap water, soil, cabbage	[61]
TPE@PDP NPs	MAT	5 fM–0.5 uM	0.9 fM	cabbage	[62]
TiO_2_@CdSe QDs	MAT	0.4 fM–4 nM	0.13 fM	cucumber, cabbage, spinach	[64]
ILu-HOFs	ICP	1 pM–100 nM	0.4 pM	lake water, cucumber juice	[65]
Ag^+^@Eu-MOF/HOF	ACE	1 fM–0.1 nM	0.398 fM	river water, tap water	[69]
Pdots	ACE	0.1 pM–10 nM	9.1 aM	fresh lettuce	[66]
CdTe/CdS/ZnSQDs	AFB1	5 pM–10 nM	0.12 pM	peanut, maize, wheat	[70]
SnS_2_ QDs	ZEN	10^−7^–500 ng/mL	0.085 fg/mL	corn juice	[71]
NHCDs	AFB1	0.01–100 ng/mL	2.63 pg/mL	corn	[73]
CdS QDs	OTA	0.05–5 nM	0.012 nM	wine, beer	[74]
MQD@SnS_2_ QDs	AFB1	0.001–100 ng/mL	0.124 pg/mL	dried fish	[75]
Co-LDH@QDs	AFB1	10^−4^–10 ng/mL	0.03 pg/mL	corn	[76]
CdTe QDs	OTA	0.0005–50 ng/mL	0.17 pg/mL	maize	[77]
CdSe@CdS QDs	OTA	1–100 ng/mL	0.89 ng/mL	lily, rhubarb	[78]
SnO_2_ QDs	ZEN	0.0005–500 ng/mL	0.16 pg/mL	pig urine, cornstarch	[82]
SnO_2_ QDs	ZEN	0.001–500 ng/mL	0.103 pg/mL	corn, wheat	[79]
NGQDs	ZEN	1 fg/mL–50 ng/mL	0.85 fg/mL	maize	[80]
Pdots	DON, ABR	5.0–50 ng/mL, 1.25 × 10^−6^–1.25 ug/mL	0.73 fg/mL, 0.38 pg/mL	wheat, milk power	[81]
SnS_2_ QDs	KAN	1 pM–10 nM	0.32 pM	milk	[84]
Pdots	streptomycin	0.5 pM–200 nM	0.12 pM	milk, honey, Yangtze River, water	[86]
Bi_2_S_3_ QDs	ENR	5 nM–25 uM, 0.5 nM–25 uM	1.59 nM (DPV), 0.13 nM (ECL)	egg	[88]
Cu NCs	ENR	0.1 pg/mL–50 ng/mL	0.06 pg/mL	milk, chicken	[89]
Au NCs	KAN	10 pM–33 uM	1.5 pM	milk	[94]
Tr-HOFs	KAN	1 nM–10 uM	0.28 nM	milk, serum	[91]
DNR-CuNCs	KAN	0.01–5 × 10^5^ pg/mL	0.18 fg/mL	milk	[96]
CdS QDs	KAN, neomycin	0.1 nM–1 uM, 1 nM–10 uM	17 pM, 0.35 nM	milk, honey	[97]
BNQDs	CAP	0.1 pM–1 uM	0.33 pM	CAP ophthalmic solution, CAP capsules, waste water, milk, honey	[98]
SnS_2_ QDs	CAP	0.005–1000 ng/mL	1.7 pg/mL	shrimp, honey	[99]
TPPE-CB [8]	CAP	10 fM–100 nM	1.81 fM	milk, honey	[92]
Cu-CdTe QDs	TC	0.01–10 ng/mL	3 pg/mL	pond water, honey, milk	[100]
OVA/ETTA	TC	0.1 pM–1 uM	42.6 fM	lake water, milk	[101]
Glu/TG-Au NCs	TC	5 fM–5 nM	2.3 fM	milk	[93]
AgBr NPs	*E. coli*	0.5–500 CFU/mL	0.17 CFU/mL	meal samples	[103]
CdS QDs	*S. aureus*	5–10^8^ CFU/mL	1 CFU/mL	pork, spinach, raw milk	[107]
Arg/ATT-AuNCs	*S. aureus*	10–10^9^ CFU/mL	1.16 CFU/mL	scallop, fish, shrimp	[108]
HOF-101@AgNPs	*E. coli O157:H7*	1–10^7^ CFU/mL, 10–10^6^ CFU/mL	0.48 CFU/mL (ECL), 2.39 CFU/mL (FL)	tap water, milk	[110]
Met-AuNCs	*VP*	10–10^7^ CFU/mL	1.23 CFU/mL	scallop, fish, shrimp, seawater, river water	[112]
PFBT Pdots	Cu^2+^	0.001–10 ng/mL	11.8 fg/mL	glycyrrhiza uralensis fisch	[114]
Au NCs	Pb^2+^	0.1 nM–0.1 mM	23 pM	farmland soil, contaminated soil	[116]
NCQDs	Pb^2+^	10 fM–10 nM	4.41 fM	tap water, river water, soil	[117]
CdTe@CdS QDs	Hg^2+^	20 aM–2 uM	2 aM	saltwater, fish, carp fish, tap water	[118]
EuS NCs	Hg^2+^	0.1–10^5^ pM	0.028 pM	fish, shellfish, shrimp	[122]
Pdots	Cd^2+^	0.01–100 ppb	0.006 ppb	ganoderma lucidum	[119]
Ag NCs	Cd^2+^, Mg^2+^	0.1 pM–10 nM, 1 pM–10 nM	45.35 fM, 0.11 pM	rice	[120]
CdSe@ZnSe QDs	melamine	10 pM–0.1 nM	3.3 pM	milk	[127]
CdSe@CdS QDs	Sudan I	0.001–500 ng/mL	0.3 pg/mL	tomato sauce, chili sauce, chili powder	[128]

## 4. Conclusions and Perspectives

The boom in nanotechnology and ECL sensing technology has fueled the development of nanoaggregates with versatile functions and roles, which have been instrumental in the creation of ECL biosensors with a markedly increased sensitivity. In recent years, the efficient, economical, and robust ECL sensing platforms engineered from these have made remarkable achievements in foodborne-contaminant assays. Given this situation, it is imperative to provide a timely and systematic summary of the latest developments in this field to promote its future commercialization. In this review, we systematically summarized the recent progress in nanoaggregate-based ECL sensors for measuring diverse foodborne contaminants, with a focus on the underlying fundamentals and representative applications, thereby offering valuable insights for the future development of this field. However, several challenges in this domain remain to be addressed as they hinder further commercial development.

Firstly, further improving the ECL efficiency of nanoaggregates remains a key goal. Current methods to improve ECL efficiency primarily rely on co-reactant accelerators and AIE natures, but these methods often introduce drawbacks involving operational complexity, insufficient stability, poor reproducibility, and elevated cost. Given these limitations, the precise adjustment of their compositions, morphologies, and structures to intrinsically optimize ECL efficiency is a vital direction for the rational design of nanoaggregates.

Secondly, developing nanoaggregate-based ECL sensors for simultaneous multi-analyte detection in real-world food samples is of great importance. Currently, most existing nanoaggregate-based ECL sensors are confined to single-analyte measurement. Proverbially, multi-analytes often coexist within a single food sample, so single-analyte detection easily results in diagnostic inaccuracy or high testing costs. Herein, the ingenious integration of wavelength/potential/space-resolved technology into these ECL sensors provides a promising solution for multiplexed analysis.

Thirdly, most nanoaggregate-based ECL sensors depend on a single-signal variation for the quantitative detection of analytes, which is severely compromised by environmental and experimental interference in complex samples. By contrast, dual-signal ratiometric ECL sensors relying on the ratio between two distinct signals to quantify analytes offer a robust alternative with superior anti-interference capability. Given this, creating ratiometric ECL sensors employing nanoaggregates is a promising strategy for breakthrough scientific outcomes.

Fourthly, current research on nanoaggregate-based ECL sensors has centered predominantly on ECL intensity changes rather than optical imaging. By comparison, the ECL imaging technique has the advantages of high throughput, direct visualization, excellent controllability, and a non-existent photothermal effect. The development of ECL imaging incorporating nanoaggregates is therefore anticipated to open a new frontier in analytical science, heralding a new era of exciting applications.

Fifthly, with the increasing development of technology, artificial intelligence (AI) is poised to substantially improve detection efficiency. Machine learning, as a core component of AI, offers powerful batch image processing to predict RGB values and analyte concentrations, which provides valuable insights for the development of nanoaggregate-based ECL biosensors assisted by smartphones. Thereupon, a comprehensive approach combining sensitive ECL detection, smartphone visualization, and machine learning-driven prediction represents an up-and-coming frontier direction in high-throughput analysis.

Lastly, nanoaggregate-based ECL sensing technology has demonstrated high sensitivity and near-zero background, but its commercialization is constrained by bottlenecks in the reliability, stability, and throughput of the sensor. Consequently, interdisciplinary studies involving a combination of nanoaggregate-based ECL sensing with other disciplines are vital to develop practical integrated detection devices to overcome the aforementioned bottlenecks and meet real-world demands.

## Data Availability

No new data were created.
